# Chemotherapy-Induced Tissue Injury: An Insight into the Role of Extracellular Vesicles-Mediated Oxidative Stress Responses

**DOI:** 10.3390/antiox6040075

**Published:** 2017-09-28

**Authors:** Chontida Yarana, Daret K. St. Clair

**Affiliations:** 1 Department of Toxicology and Cancer Biology, University of Kentucky, Lexington, KY 40536, USA; cya224@g.uky.edu; 2 Faculty of Medical Technology, Mahidol University, Salaya 73170, Thailand

**Keywords:** chemotherapy, oxidative stress, extracellular vesicles, superoxide dismutase

## Abstract

The short- and long-term side effects of chemotherapy limit the maximum therapeutic dose and impair quality of life of survivors. Injury to normal tissues, especially chemotherapy-induced cardiomyopathy, is an unintended outcome that presents devastating health impacts. Approximately half of the drugs approved by the Food and Drug Administration for cancer treatment are associated with the generation of reactive oxygen species, and Doxorubicin (Dox) is one of them. Dox undergoes redox cycling by involving its quinone structure in the production of superoxide free radicals, which are thought to be instrumental to the role it plays in cardiomyopathy. Dox-induced protein oxidation changes protein function, translocation, and aggregation that are toxic to cells. To maintain cellular homeostasis, oxidized proteins can be degraded intracellularly by ubiquitin-proteasome pathway or by autophagy, depending on the redox status of the cell. Alternatively, the cell can remove oxidized proteins by releasing extracellular vesicles (EVs), which can be transferred to neighboring or distant cells, thereby instigating an intercellular oxidative stress response. In this article, we discuss the role of EVs in oxidative stress response, the potential of EVs as sensitive biomarkers of oxidative stress, and the role of superoxide dismutase in attenuating EV-associated oxidative stress response resulting from chemotherapy.

## 1. Introduction

Improved cancer treatment has raised the five-year survival rate of adult cancer patients from 49% in the late 1970s to 69% between 2005 and 2011. In childhood cancer patients, the survival rate is even better, having improved from 58% in the 1970s to 83% by 2011. However, these gains have been accompanied by increased risks. The longer a cancer patient lives, the greater the possibility that complications from cancer treatment will occur. Complications can arise, in part, from oxidative stress-induced noncancerous tissue damage, which decreases the quality of life of cancer survivors. Treatment-related side effects such as cardiotoxicity are the third leading cause of death in adult and childhood cancer survivors. Recurrence and secondary tumors are the first and second leading causes of death, respectively [1]. The American Cancer Society has predicted that there will be 19 million long-term cancer survivors in the U.S. by 2024. This immense number of survivors will necessitate an emphasis on preventive strategies that preserve normal tissues while killing cancer cells.

Currently, 215 drugs have been approved by the Food and Drug Administration to treat cancer. At least 50% of them can induce oxidative stress, among them anthracycline, cyclophosphamide, cisplatin, busulfan, mitomycin, fluorouracil, cytarabine, and bleomycin [2]. Excessive reactive oxygen species (ROS)/reactive nitrogen species (RNS) that reach the threshold of antioxidant capacity of the cells, as well as the accumulation of oxidized biomolecules that overwhelms biomolecular quality controls of cells cause cytotoxicity to both cancer and normal cells. To combat against oxidative stress-induced cytotoxicity, cells have evolved several pathways to remove oxidized toxic molecules including proteasome-mediated degradation and autophagy, as well as removing the toxic molecules in the form of extracellular vesicles.

Extracellular vesicles (EVs) are membranous encapsulated structures that are released from almost all cell types. EVs are heterogeneous in size, content, and biogenesis. They are generally classified into three types based on their biogenesis: exosomes, microvesicles, and apoptotic bodies. EVs serve as vehicles that transfer bioactive molecules between cells and, thus, mediate cell-cell communication during cellular stress.

This review focuses on the effects of chemotherapy on oxidative stress-induced tissue injury as exemplified by Doxorubicin (Dox); how protein quality control is affected by Dox; the role of extracellular vesicles in oxidized protein removal; and the relationship between extracellular vesicles and superoxide dismutase, a major antioxidant that protects cells from oxidative damage.

## 2. Mechanisms of Chemotherapy-Induced Cardiomyopathy

Decades-long research that has explored the mechanisms by which Dox cause cardiomyopathy via multiple pathways has been extensively reviewed [3]. Dox-induced cardiomyopathy engages not only cell death-related signaling pathways in cardiomyocytes themselves, but also the intercellular communication leading to tissue inflammation and maladaptive remodeling.

The generation of ROS and RNS is an initial event of Dox-induced cardiac tissue injury. Mitochondria are major subcellular organelles affected by Dox, since Dox has 300–1000 fold higher affinity to bind cardiolipin, a major component of inner mitochondrial membrane, compared to other anionic phospholipids [4]. The redox cycling of quinone-Dox to semi-quinone radical—formed by diverting one electron from NADH dehydrogenase at complex I and donating the electron to molecular oxygen—gives rise to superoxide radical (O_2_•^−^) formation [5]. O_2_•^−^ can also be generated by other enzymatic reactions such as cytochrome P450 reductase, xanthine oxidase, and uncoupled nitric oxide synthase, as well as non-enzymatic reactions from redox cycling of Dox-Fe^3+^ complex [6].

Manganese superoxide dismutase (MnSOD) is the major enzyme responsible for mitochondrial O_2_•^−^ removal, yielding hydrogen peroxide (H_2_O_2_) as a product. H_2_O_2_ can be further detoxified by catalase and glutathione peroxidase. However, excessive superoxide radicals can react with other ROS and RNS such as H_2_O_2_ and nitric oxide radical (•NO) to form more highly reactive ROS/RNS.

Aconitase is a tricarboxylic acid cycle enzyme responsible for catalyzing the conversion of aconitate to isocitrate [7]. Aconitase contains iron-sulfur clusters that are sensitive to O_2_•^−^ inactivation, which results in the release of Fe^2+^ from the enzyme [8]. O_2_•^−^ can also induce the release of Fe^2+^ from Ferritin, an intracellular iron storage protein [9]. Fe^2+^ further catalyzes the interaction of O_2_•^−^ with H_2_O_2_, leading to hydroxyl radical (•OH) generation. The interaction of O_2_•^−^ with •NO generates peroxinitrite (ONOO^−^). ONOO^−^ and •OH are highly reactive and can damage biomolecules such as lipids, proteins, and nucleic acids.

Unsaturated fatty acids, major components of cellular membranes, contain carbon-carbon double bonds, making them susceptible to ROS/RNS attack. Two major ω_6_ lipid peroxidation products derived from non-enzymatic ROS/RNS attack are malondialdehyde (MDA) and 4-hydroxynonenal (4HNE). Among the major lipid peroxidation products, 4HNE is the most toxic molecule. 4HNE rapidly reacts with thiols and amino groups of amino acids, leading to 4HNE protein adduction. It has been shown that protein-bound 4HNE forms early after Dox treatment. In cardiac mitochondria, protein-bound 4HNE forms as early as 3 h following treatment, and rises to its highest level at 6 h post-treatment. An increase in 4HNE-bound proteins occurs in mitochondria prior to occurring in cytoplasm and the nucleus [10]. A high level of 4HNE leads to the inactivation of the adducted proteins. Our recent study showed that during Dox treatment, 4HNE-adducted proteins in heart tissues that are involved in the electron transport chain, such as succinate dehydrogenase A (SDHA), dihydrolipoamide dehydrogenase (DLD), ATP synthase subunit β (ATP5B), and NADH dehydrogenase [ubiquinone] iron-sulfur protein 2 (NDUFS2), led to mitochondrial respiratory dysfunction and a shift of energy production to glycolysis [11]. Furthermore, 4HNE adduction to mitochondrial protein, as in mitochondrial apoptotic inducing factor 2 (AIFM2), can alter the protein function from oxidoreductase to pro-apoptotic protein, leading to cardiomyocyte cell death [12].

## 3. Oxidized Protein Removal Pathways

Cells contain several protein quality control (PQC) mechanisms that maintain physiological homeostasis against highly toxic oxidized protein accumulation. Those mechanisms involve protein unfolding via endoplasmic reticulum, intracellular protein degradation via the ubiquitin-proteasome pathway, autophagy-lysosomal pathways, and extracellular vesicles release [13,14] PQC is highly regulated by cellular redox status. A growing body of evidence shows that Dox affects PQC in cardiomyocytes, leading to the accumulation of oxidized proteins and eventually cell death [15,16,17].

### 3.1. Protein Unfolding System

Protein oxidative modification involves a disturbance of protein folding, resulting in aberrant protein conformations that affect protein function, stability, and solubility [18]. Protein unfolding occurs in the organelle endoplasmic reticulum (ER), where it serves as a first-line response against toxic oxidized proteins. Protein unfolding is initiated by stress-sensor proteins on the ER membrane, activating transcription factor 6 (ATF6), inositol-requiring enzyme 1 (IRE1), and protein kinase RNA-like ER kinase (PERK). ATF6 and IRE1 activation initiates an adaptive response by upregulating 78 kDa glucose-related protein (GRP78), an ER chaperone protein responsible for regulating protein folding. PERK activation regulates negative feedback control by inhibiting protein translation and thus alleviating the ER workload [19]. However, excessive ER stress initiates the apoptotic pathway mediated by caspase-12, c-Jun NH2-terminal kinase (JNK), and C/EBP homologous protein (CHOP). A histological study of endomyocardial biopsies from patients experiencing DOX-induced cardiotoxicity revealed extensive ER dilation [20], suggesting that ER stress is a major mechanism of DOX-induced cardiotoxicity. Recently, a study in mice revealed that DOX activated ATF6 and IRE1 but failed to upregulate GRP78 gene expression, leading to a shift from ER adaptive stress response to apoptosis response [21].

### 3.2. Ubiquitin-Proteasome System (UPS)

The Ubiquitin-proteasome system (UPS) is responsible for removing cytosolic oxidized proteins, which are ubiquinitated by three classes of enzymes working in concert. Ubiquitin activation enzyme (E1) activates and transfers ubiquitin to ubiquitin conjugation enzyme (E2). Ubiquitin ligase (E3) brings in the target substrate and transfers ubiquitin from E2 to lysine residues of the substrate. The polyubiquitinated proteins at lysines are subsequently recognized and degraded by the 26s proteasome. Alternately, oxidized proteins without ubiquitination can be degraded by the 20s proteasome. UPS is highly regulated by cellular redox status. Mild oxidative stress upregulates ubiquitination machinery and proteasome activity. However, severe oxidative stress inhibits proteasome activity while it spares the ubiquitination systems, leading to the accumulation of oxidized-polyubiquitinated proteins [22]. Dox is known to cause massive protein oxidation and ubiquitination. However, the role of Dox in UPS remains controversial. A study of H9c2 cardiomyoblasts treated with 3 µM Dox for 24 h and tumor-bearing mice treated with a cumulative dose of Dox 20 mg/kg revealed that Dox inhibits proteasome activity while it stimulates protein ubiquitination by increasing E3 ligase expression [23]. However, another study using a lower concentration of the therapeutic dose (0.1–5 µM) found that Dox upregulates E3 ligase, the C-terminal of heat shock protein cognate 70-interacting protein (CHIP), and heat shock protein 70, as well as activates 20s proteasome [24]. Nevertheless, proteasome function relies heavily on protein unfolding for the translocation of the protein into the proteasome lumen to occur [25]. Dox that causes extensive oxidized protein misfolding and aggregation might compromise the UPS system despite the enhanced proteasome activity.

### 3.3. Autophagy

Autophagy, literally self-digestion, contributes to the degradation of cytosolic protein aggregates and dysfunctional organelles. Damaged components of autophagy are trafficked to lysosome for degradation. This process can be mediated by three different pathways; macroautophagy, microautophagy, and chaperone-mediated autophagy (CMA). Macroautophagy is characterized by the sequestration of the cargoes by double-membrane structures, which progress to autophagosome formation and subsequently fusion to lysosome for degradation. The process of macroautophagy initiates with the activation of the unc-51-like autophagy activating kinase 1 (ULK-1) complex, which further activates Beclin-1 and the vesicle-mediated vacuolar protein sorting (VPS34) complex—the two proteins that mediate phagophore formation. Subsequently, autophagy-related gene (ATG) proteins 5, 12, and 16 are recruited to the phagophore membrane and extend the phagophore. The cytosolic form of microtubule-associated protein 1A/1B-light chain 3 (LC3-I) is then lipidated with phosphatidylethanolamine to form LC3-II, which is also recruited to the phagophore membrane. Ubiquitinated damaged protein substrate, along with p62/SQSTM1, interacts with LC3-II, all of which are then engulfed by phagophore to form an autophagosome. The final process is the fusion of the autophagosome with a lysosome and the degradation of the autophagosome cargoes by lysosomal enzymes. Microautophagy directly engulfs the cytosolic component by the invagination of the lysosomal membrane. CMA requires heat shock-cognate protein of 70 KDa (HSC70) to recognize proteins that contain pentapeptide sequence KFERQ. HSC70-bound KFERQ-containing protein will then bind to lysosome-associated membrane protein type 2A (LAMP-2A) on the lysosomal membrane, which translocates the target protein into the lysosomal lumen for degradation. Blocking of autophagic flux leads to an accumulation of oxidized proteins and organelles that are toxic to the cells.

Dox has been shown to disrupt macroautophagy and CMA. The process of autophagy dysregulation following Dox exposure has been extensively reviewed [16,26,27]. Dox promotes autophagosome formation by increasing LC3-II levels and ATG protein expression [28]. Dox-derived lipid peroxidation product 4HNE is also a potent activator of aldehyde-induced autophagy [29]. Our previous study found that p53 is a key player that drives JNK1-mediated autophagosome formation, and that p53 knockout mice are protected from Dox-induced oxidative stress in mitochondria [30]. Recently, Bartlett and colleagues reported that Dox inhibits vacuolar ATPase (VATPase), a proton pump on the lysosomal membrane that maintains the level of acidic pH required for lysosomal degradation. Furthermore, the same group also found that Dox suppresses the expression of transcription factor EB, which is a key protein governing lysosomal content and function [31]. The lysosome blocking effect leads to an accumulation of undegraded autolysosomes, resulting in additional ROS production and cardiac injury [15].

### 3.4. Mitophagy

Healthy mitochondria are required for ATP generation that supports cardiac contractility. Mitophagy, a major influence on the quality of mitochondria, relies on autophagy machinery. Two mitophagy pathways have been described in cardiomyocytes—phosphatase and tensin homolog-induced putative kinase 1 (PINK1)/E3 ubiquitin-protein ligase Parkin-mediated, and BCL2 interacting protein 3-like (Bnip3L)/Nip3-like protein X (NIX)-mediated pathways. The PINK1/Parkin pathway is triggered by mitochondrial membrane depolarization, which promotes the stability of PINK1, a serine/threonine kinase located on the mitochondrial outer membrane. PINK1 then attracts and binds to Parkin, which subsequently ubiquitinates proteins responsible for mitochondrial movement cessation and mitochondrial fragmentation maintenance. This ubiquitination process targets the damaged mitochondria for lysosome degradation [32]. In contrast to the PINK1/Parkin pathway, the Bnip3L/NIX pathway does not require mitochondrial membrane depolarization, but involves phosphorylation near the LIR motif of Bnip3L, which makes it a docking site for LC3-II on the autophagosomal membrane.

Dox affects PINK1/Parkin and Bnip3L/NIX mitophagy in different ways. Dox suppresses PINK1/Parkin mitophagy by reducing PINK1 mitochondrial translocation. Hoshino and colleagues reported that Dox-induced p53 activation leads to the interaction and sequestration of PINK-1 by p53 [33]. In contrast, Dox enhances Bnip3L/NIX mitophagy by inducing the mitochondrial translocation of Bnip3L, which leads to mitochondrial depolarization and Parkin recruitment [34]. However, cytosolic sequestration of PINK1, the Parkin partner, may blunt the effect of the Bnip3L pathway. Although Dox can either suppress or activate the upstream process of mitophagy, Dox-induced lysosome dysfunction, which blocks the end process of mitophagy, could lead to the retention of undigested damaged mitochondria.

## 4. Extracellular Vesicles

“Extracellular vesicles” is a general term that applies to membranous vesicles released from cells into the extracellular space. EVs are composed of a lipid bilayer that encapsulates a variety of biomolecules, including proteins, DNAs, RNAs, and carbohydrates, as well as subcellular organelles such as mitochondria [35,36]. EV cargoes are resistant to enzymatic degradation due to the lipid bilayer shelter. EV stability has been evaluated in various conditions. Serum EVs are stable up to 24 h at room temperature, 1 week at 4 °C, and fewer than three freeze-thaw cycles. Prolonged storage beyond the indicated time period and too many freeze-thaw cycles will cause EV rupture and content leakage [37]. Milk-derived EVs can withstand acidic pH (pH = 2.0), a short boiling temperature (105 °C, 15 min), and snap freezing in liquid nitrogen. However, the same conditions significantly degrade cell-derived EVs by 90%, 90%, and 70%, respectively [38].

Despite EV stability ex vivo, the half-life of EVs in vivo is only 2 min, due to a rapid clearance by interaction with other recipient cells [39]. The ability of EVs released from one cell to interact with other cells makes EVs a mediator of cell-cell communication. EVs express docking proteins on their surface, which could be recognized by the recipient cells. The message from one cell to another could be achieved by the interaction of EVs with the cognate receptor on the recipient cell surface, the fusion of the EV membrane with the recipient plasma membrane, or phagocytosis and delivery of the EV contents into the recipient cytosol [40].

EVs are heterogeneous in size, content, and biogenesis. Exosomes are the smallest EVs (50–100 nm) and originate from the invagination of late endosomal membranes forming multiple intraluminal vesicles (ILVs) called multivesicular bodies (MVBs). Those ILVs become exosomes when they are released by the fusion of the MVB membrane with the plasma membrane. Microvesicles (200–1000 nm) are derived from outward budding of the plasma membrane. Apoptotic bodies (>1000 nm) are generated by apoptotic cell fragmentation [41].

### 4.1. Exosome Biogenesis

MVB formation requires machinery that not only sorts proteins into the endosomal membrane, but also sorts proteins involved in changing membrane curvature and vesicle scission. Endosomal sorting complexes required for transports (ESCRTs: ESCRT-0 to ESCRT-III) are the best-known protein complexes that govern protein loading into exosomes. ESCRT-0 binds to ubiquitinated protein cargo and distributes it to the MVB membrane. ESCRT-I associates with ESCRT-0 and ubiquitinated proteins, and helps the membrane translocation. ESCRT-II is a bridge that connects ESCRT-I to ESCRT-III, which is key to membrane budding and vesicle formation. In the final step, vacuolar protein sorting-associated protein 4 (VPS4) complex dissociates ESCRT-III, thereby recycling the ESCRTs machineries [42]. However, ESCRT is dispensable to MVB generation in some cell types. For example, oligodendroglial cells utilize neutral sphingomyelinase to generate ceramide-forming lipid-raft microdomain and endosomal membrane budding [43]. In lung cancer cell lines after radiation, p53 is activated, leading to the transcription of tumor suppressor-activated pathway-6 (TSAP6), a transmembrane protein involved in MVB formation [44,45]. The p53-dependent MVB formation provides a link to how DNA damage promotes exosome release.

Protein sorting into MVBs can be alternatively mediated by HSC70, a key protein that binds to oxidized protein in CMA. However, the interactions of HSC70 with the endosomal membrane and with the lysosomal membrane are different. HSC70 relies on the electrostatic force of its basic residues, which attaches to acidic phospholipids on the cytosolic part of the endosomal membrane rather than binding to LAMP2A [46].

Exosome biogenesis and autophagy are closely related. MVB cargoes are at a crossroads that could head to degradation by lysosome fusion or be released by plasma membrane fusion. The final destination of MVBs is governed by the Rab family of small GTPases. Rab7 directs MVBs to fuse with lysosome, while Rab27A directs them to fuse with plasma membranes [47,48]. Thus, if one pathway is blocked, the cargo will pass to the other pathway. For example, ESCRT depletion in *Caenorhabditis elegans* enhances autophagic flux, which rescues cells from abnormal endosome buildup [49]. Conditions that promote autophagy divert MVBs to the lysosome rather than the plasma membrane, thus inhibiting exosome release [50]. On the other hand, autophagy defects, such as in neurogenerative diseases, promote the release of oxidized protein aggregates via exosomes [51]. In addition, chemical or genetic inhibition of phosphoinositide kinase PIKfyve, a key enzyme in membrane trafficking of autophagy, promotes the release of exosomes that contain proteins related to autophagy [52].

### 4.2. Microvesicles (MVs) Biogenesis

Microvesicles are EVs that originate in the plasma membrane. The formation of microvesicles involves the rearrangement of plasma membrane phospholipids and the reorganization of the underlying cytoskeleton. An initial factor that promotes microvesicle formation is an increase in intracellular Ca^2+^, which is driven by oxidative stress [53]. Increased Ca^2+^ inhibits inward aminophospholipid transloase (flippase) and activates outward translocase (floppase). This aberrant phospholipid translocation leads to an externalization of phosphatidyl serine (PS), a characteristic of general MVs [54]. However, some MVs do not have PS on the outer membrane leaflet [55]. Ca^2+^ overload also activates caspases that lyse the cytoskeleton, making it dissociate from the plasma membrane at the budding site. MV pinch-off involves a cascade of signaling that is triggered by GTP-binding protein ADP-ribosylation factor 6 (ARF6). ARF6 activates phospholipase D, leading to phosphatidic acid generation, which recruits extracellular signal-regulated kinase (ERK) to the plasma membrane. ERK subsequently phosphorylates myosin light-chain kinase (MLCK), which further phosphorylates the myosin light chain (MLC). This signaling cascade results in actomyosin-based contraction at the neck of the bud, leading to MV release [56].

### 4.3. Apoptotic Bodies Biogenesis

Unlike exosomes and MV formation, apoptotic bodies are generated by dead cells. However, a common pathway observed in both apoptotic body formation and MV formation is MLC phosphorylation, which is responsible for membrane protrusion. Instead of the ARF-6-mediated process, membrane blebbing of apoptotic cells initiates caspase-3 activation, which cleaves and activates Rho-associated coiled-coil protein kinase 1 (ROCK-1), an enzyme that phosphorylates MLC [57]. Because caspase activation causes DNA cleavage and cytochrome c release from mitochondria, the remnants of this process, such as nuclear DNA and mitochondrial fragments, can be found in apoptotic bodies.

### 4.4. EVs Serve as a Bypass Highway for Oxidized Proteins Removal during Dox-Induced Cardiotoxicity

**Figure 1:**
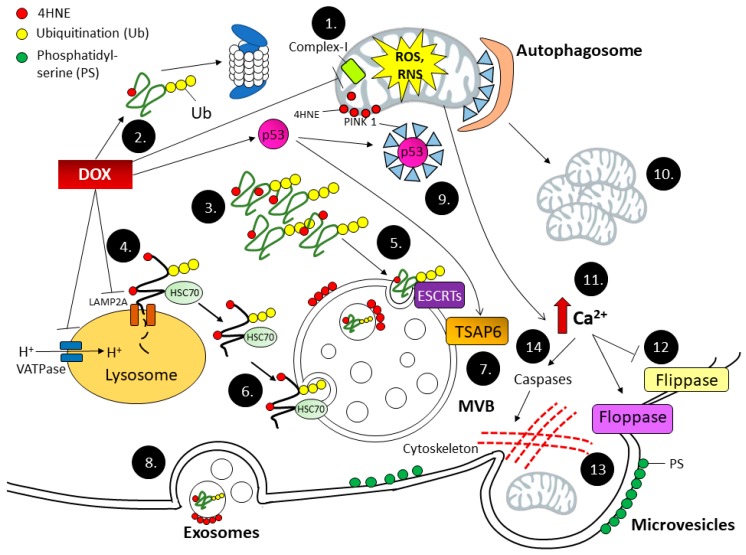
Dox inhibits intracellular protein quality control pathways while promoting extracellular vesicle release.

Dox generates extensive quantities of oxidized and ubiquitinated proteins while it disrupts multiple pathways of protein quality control as discussed above. Because EV biogenesis and protein quality control pathways are interconnected, EVs serve as an alternative pathway to remove toxic proteins from cells during Dox-induced oxidative stress. Indeed, Dox can promote EV release in several ways. Dox activation of p53 can increase MVB production via TSAP6 activation [44,45]. In addition, Dox disrupts Ca^2+^ homeostasis in cardiomyocytes by interfering with the electron transport chain, leading to the potential collapse of the mitochondrial membrane [58]. Inability to maintain the mitochondrial membrane potential inhibits Ca^2+^ influx into mitochondria, which results in an elevation of cytosolic Ca^2+^, a key event that induces MV release. Lipid peroxidation by Dox may affect membrane curvature and promote EV formation, as evidenced by the photooxidation of the artificial lipid membrane, inducing the alteration of its physical property, in which the hydroperoxyl group increases the area of the lipid membrane, thus forming membrane budding [59]. The effect of Dox on protein quality control pathways as well as EV biogenesis is summarized in Figure 1.

Dox redox cycling at complex I leads to excessive ROS and RNS generation in mitochondria, which leads to biomolecule oxidation (1). 4HNE is a reactive lipid peroxidation product that can adduct to proteins and inactivate protein function (2). Oxidized proteins as well as 4HNE-adducted proteins are subject to degradation by the protein quality control pathways, including protein unfolding, the ubiquitin-proteasome pathway, and autophagy. Severe protein oxidation due to Dox insults generates bulky misfolded proteins, which overwhelm proteasome degradation, thus forming protein aggregates (3). Autophagy is a major pathway to remove and recycle protein aggregates, as well as damaged organelles such as mitochondria. Dox inhibits VATPase, a proton pump that maintains lysosomal pH, as well as LAMP2A, a membrane transporter of oxidized protein into the lysosomal lumen for degradation (4). The lysosomal dysfunction leads to an accumulation of oxidized proteins, which can be alternatively sorted into MVBs by ESCRT complexes (5), as well as HSC70 (6). Dox-induced DNA damage activates p53, which further upregulates TSAP6, an endosomal membrane protein responsible for MVB formation (7). Exosomes are generated by the fusion of MVBs with the plasma membrane, which releases intraluminal vesicles to the extracellular space (8). Dox blocks mitophagy via p53-mediated PINK1 sequestration into cytosol, thus preventing autophagosome formation (9). Dysfunctional mitochondria that cannot be cleared by autophagy accumulate (10). Meanwhile, mitochondrial dysfunction induces Ca^2+^ retention in the cytoplasm (11), which in turn inhibits flippase and activates floppase (12). PS externalization occurs due to floppase activity (13). Increase in cytosolic Ca^2+^ also activates caspases, which cleaves the cytoskeleton and dissociates the membrane from the underlying structure, forming MVs (14).

Since the mechanisms by which Dox promotes EV release are based primarily on oxidative stress, it follows that this concept can be applied to other chemotherapeutic agents that generate ROS/RNS as well. In fact, Hall and colleagues found a significant increase in endothelial-derived MVs in the blood of multiple myeloma patients treated with cyclophosphamide, thalidomide/lenalidomide, and dexamethasone comparing to baseline [60]. In addition, cancer cells exploit EVs to remove intracellular chemotherapeutic drugs and promote chemo-resistant phenotypes. Recently, Muralidharan-Chari and colleagues reported that human pancreatic cancer cells release MVs to expel Gemcitabine—a chemotherapy that inhibits DNA synthesis. The ability of the cells to release MVs for Gemcitabine clearance correlated with the degree of drug resistance, and the inhibition of MV release sensitized the cells to Gemcitabine [61].

### 4.5. EVs as a Biomarker for Oxidative Stress

**Figure 2:**
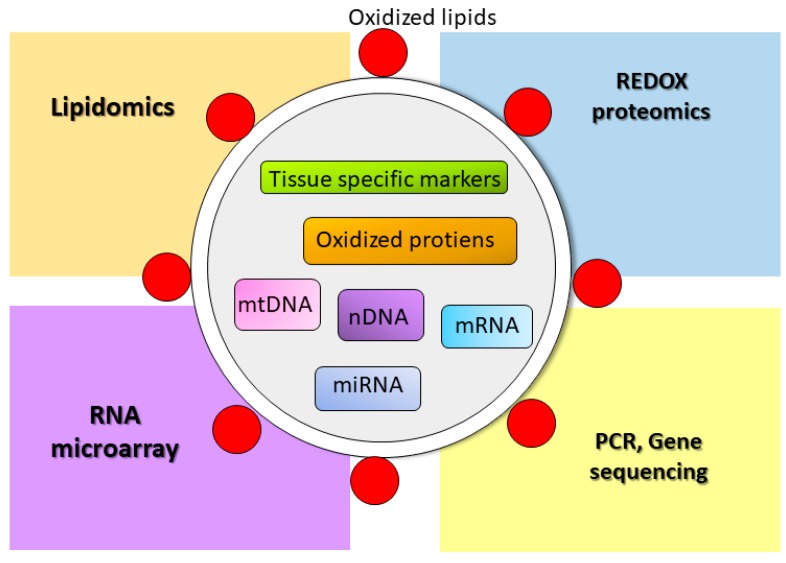
Candidate molecules in extracellular vesicles (EVs) for oxidative stress biomarkers.

For many reasons, EVs are more attractive for biomarker discovery than conventional serum biomarkers. In addition to their stability and ability to carry many kinds of biomolecules, as discussed above, EVs are abundant in various body fluids, including blood, urine, saliva, milk, lymph, ascites, and amniotic fluids. EVs that are released from viable tissues in the form of exosomes and MVs serve as useful tools of early markers of tissue injury that are detectable prior to cell death. Since EVs are highly associated with tissue redox status, a study of oxidatively modified molecules will be beneficial for biomarker discovery of oxidative stress-mediated diseases, especially in chemotherapy-induced tissue injury, where early cardioprotective intervention is a goal. Figure 2 shows potential EV cargo that could be used as a biomarker for oxidative stress and potential methods to identify those markers.

Cellular oxidative stress leads to the production of oxidatively modified molecules such as oxidized lipids, oxidized proteins, and mitochondrial DNA (mtDNA) fragments, which are sorted to EVs for removal. Those oxidized molecules as well as apoptotic nuclear DNA and tissue specific proteins, mRNA and miRNA, can identify the origin as well as the oxidative status of the releasing cells. Several methods, such as lipidomics, REDOX proteomics, RNA microarray, PCR, and gene sequencing, can be applied to discover the potential molecule to be used as a certain disease biomarker.

### 4.6. Role of EVs in Oxidative Stress Response

EVs play a key role in mediating cell-cell communication. They shuttle bioactive lipids, mRNA, and miRNA, as well as signaling proteins that drive biological changes in recipient cells. EVs have advantages over other types of intercellular communication because the signaling molecules inside them are protected from enzymatic degradation. Thus, the messages can be delivered to sites away from the releasing spot. The messages can also be delivered with high specificity to recipient cells via receptor-mediated endocytosis [62]. Intercellular communication via EVs is critical to the progression of many oxidative stress-related diseases, among them: cancer-microenvironment crosstalk—a key event in cancer progression and metastasis [63]; spreading toxic protein aggregates in neurodegenerative diseases [64]; and carrying damage and pathogen-associated molecular patterns (DAMPs and PAMPs), autoantigens, and proinflammatory cytokines to activate immune cells in inflammatory diseases [65]. In this review, we discuss the role of EVs in the context of oxidative stress.

Table 1 summarizes the effect on recipient cells of EVs released under different oxidative stress conditions. Briefly, oxidative stress conditions affect the contents of EVs. Those contents can induce oxidative stress response in recipient cells either by helping to protect the releasing cells against further injury or by exacerbating the injury. Although EVs serve as a compensatory mechanism that deal with proteotoxicity, the consequences of proteotoxic cargo transference to neighboring cells can be detrimental. Malik et al. showed that ROS induction in adult rat cardiomyocytes treated with ethanol or brief hypoxia/reoxygenation condition leads to HSP60-containing exosome release, as HSP60 causes cardiomyocyte apoptosis [66]. However, the spreading of HSP60 by exosomes to neighboring cells could activate Toll-like receptor 4 (TLR4)-mediated apoptosis in recipient cells [67]. In contrast, Eldh et al. reported that mRNA profiling in exosomes changes when mast cells are treated with H_2_O_2_ and the exosomal mRNA help mast cells tolerate higher doses of H_2_O_2_ [68]. Retinal pigment epithelial cells treated with ethanol release exosomes that contain vascular endothelial growth factor receptor as well as the mRNA of the protein and the exosomes promotes angiogenesis [69].

EVs are well-known to function as an immunomodulators. EVs can present cellular peptide antigens on their surface with major histocompatibility complex (MHC) class I and class II molecules. The peptide antigen can activate T-cells directly or indirectly via transferring to antigen-presenting cells. Accumulating evidence indicates that EVs released under oxidative stress conditions can stimulate immune cells. Stress EVs which are generated by treating HEK293 cells with Ca^2+^ ionophore (A23187) or by treating synthetic EVs with 15-lipoxygenase and Fenton reaction contain lipid peroxidation products on their surfaces. These stress EVs activate TLR4-mediated NFκB signaling and pro-inflammatory cytokine release from macrophages [70]. Plasma of mice fed a high-fat diet releases MVs containing oxidized mtDNA, which can induce TNFα and IL-6 production through TLR9 activation [71]. Oxidative stress in an alcoholic hepatitis model contains mtDNA in hepatocyte-derived MVs, which can also activate TLR9 and promote neutrophilic inflammation [72]. Another alcoholic hepatitis model presented hepatocyte release exosomes with miR-122, a liver-specific miRNA. The miR-122 in exosomes is biologically active in that it can inhibit heme oxygenase-1, making the monocyte more sensitive to LPS-induced TNFα and IL-1β production [73]. Mouse and human hepatocytes treated with palmitate to induce lipotoxicity release MVs that express TNF-related apoptosis-inducing ligand (TRAIL) on their surface, which were recognized by death receptor 5 on macrophages, resulting in IL-6 release [74]. Immune cells can receive EV-induced cytokine production signals from non-immune cells. Wang et al. recently reported that myocardial infarction induces macrophages to release exosomes containing miR-155, which can transfer to cardiac fibroblasts. This leads to the downregulation of the suppressor of cytokine signaling 1 (SOCS1)—a negative regulator of cytokine signaling—thus promoting cardiac inflammation [75]. However, a recent study from Cambier et al. found that the most abundant RNA in EVs released during myocardial infarction from cardiosphere-derived cells are Y RNA fragments, small non-coding RNAs. The delivery of Y RNA to macrophages upregulates IL-10 and protects cardiac tissue from ischemia/reperfusion injury [76].

## 5. Role of Superoxide Dismutase in EV-Associated Oxidative Stress Response

Superoxide dismutase (SOD) is a compartmentalized ROS detoxifying enzyme that catalyzes the dismutation of O_2_•^−^ to H_2_O_2_ and O_2_ [77,78]. Three isoforms of SOD have been characterized in mammals. SOD1 (CuZnSOD) is a homodimer that contains Cu and Zn at the catalytic cite. CuZnSOD is located ubiquitously in cytoplasm, the mitochondrial intermembrane space, nucleus, and lysosome. SOD2 (MnSOD) is a Mn-containing homotetramer enzyme with a mitochondrial localization sequence that makes it reside in the mitochondrial matrix. SOD3 (ECSOD) is a Cu- and Zn-containing homotetramer enzyme localized on the cell surface by the interaction of its C-terminal region with heparin and other extracellular matrix proteins [79,80]. ECSOD can be detected in body fluids such as plasma, lymph, ascites, synovial fluid, and cerebrospinal fluids [81,82]. SOD plays a role in cell signaling under physiological as well as pathological conditions. Recent studies have begun to reveal the interplay between SOD and the EV pathway. A discussion of the relationship between SOD and EVs in the context of EV-associated oxidative stress response follows.

### 5.1. SOD1

The *SOD1* gene is located on chromosome 21 (21q22.1 region). The SOD1 activity of a person with Down syndrome (trisomy 21) is 50% higher than in the normal population. Although the mechanism of how SOD1 promotes the Down syndrome phenotype is unknown, one possible explanation is the out proportion of SOD1 activity over glutathione peroxidase 1 activity, which leads to the overproduction of H_2_O_2_—a key senescence mediator [83]. The well-established link between SOD1 and human disease is typified by the development of amyotrophic lateral sclerosis (ALS), a neurodegenerative disease characterized by the progressive loss of motor neurons in the corticospinal tract involving the brain, brainstem, and spinal cord. SOD1 mutations account for 1–4% of sporadic ALS patients and 20% of familial ALS patients [84]. SOD1 mutation interferes with protein stability and folding, which leads to aggregate formation [85]. The aggregated SOD1 can be transferred to other neurons by contiguous propagation to the neighboring neurons or by the network propagation of synaptic transmission [86]. Grad and colleagues found that aggregated SOD1 can be released extracellularly with or without exosome association. The free SOD1 or the exosome-associated SOD1 can be taken up by recipient cells. However, many questions remain to be clarified in exosome-mediated SOD1 transfer. For instance, how is aggregated SOD1 sorted into MVBs, and is the SOD1 in the exosomes degraded in the recipient cells by the lysosomal pathway, or does it cause proteotoxicity in the recipient cells?

### 5.2. SOD2

SOD2 is located exclusively in mitochondria. Its major role is to protect mitochondria from the damaging effects of O_2_•^−^ (reviewed comprehensively in [87]). Complete SOD2 knockout in mice is lethal in early post-natal life due to dilated cardiomyopathy [88]. Life-long heterozygous deletion of SOD2 in mice reduces 50% of SOD2 activity and leads to oxidative damage in all tissues, as evidenced by the elevation of 8-OHdG in nuclear and mtDNA. Although aging is not affected by partial SOD2 deletion, the mice have a two-time higher incidence of developing tumors and 80% have multiple tumors, including lymphoma, hemangioma, adenocarcinoma, and pituitary adenoma [89].

Excessive ROS generation from mitochondria is a major mechanism of Dox-induced cardiotoxicity. Our lab previously discovered that SOD2 overexpression protects heart tissue from Dox-induced mitochondrial injury [90]. In those animals, mitochondrial complex I is spared from O_2_•^−^ insult [91]. Mitochondrial iron is a critical mediator of cardiotoxicity from Dox. Dexrazoxane, an iron-only chelator that is FDA-approved for use as a cardioprotective agent against Dox toxicity, is more effective than other iron chelators since it can reduce mitochondrial iron [92]. SOD2 affects mitochondrial iron in a manner similar to the effect of dexrazoxane. In mice, the loss of SOD2 from erythroid progenitor cells accumulates mitochondrial iron, leading to protein oxidation and membrane deformity—a phenotype similar to ringed sideroblast in hemolytic anemia [93].

As discussed above, oxidative stress and impaired protein quality control drive EV release, which in turn can promote further tissue damage and tissue inflammation. Although the direct effect of SOD2 on EV oxidative stress response has not yet been investigated, we speculate that SOD2, which limits ROS production, can reduce oxidative stress-related tissue injury from the first wave of chemotherapy insult, as well as prevent the second wave of injury mediated by damaging cell-derived EVs.

### 5.3. SOD3

SOD3 is a secretory CuZnSOD. The human *SOD3* gene is localized on the 4p-q21 region of chromosome 4 [94]. It shares 60% homology with the *SOD1* gene, but very low homology with the *SOD2* gene. Human SOD3 mRNA is highly enriched in certain tissues, including heart, placenta, pancreas, and lung, and less enriched in kidney, skeletal muscle, liver and brain tissue [95]. As it is located on cell surfaces and in the extracellular matrix, SOD3 plays a major protective role against oxidative damage in the extracellular environment. •NO in the extracellular environment is crucial for maintaining cardiovascular homeostasis. •NO regulates vascular tone; inhibits platelet aggregation and leukocyte adhesion; and prevents vascular inflammation. O_2_•^−^ generated by Xanthine oxidase, NADPH oxidase, and uncoupled endothelial nitric oxide synthase interacts with •NO forming ONOO^−^, which in turn oxidizes various biomolecules, leading to vascular dysfunction. SOD3 promotes the bioavailability of •NO by removing the inhibiting O_2_•^−^, thus helping to maintain vascular function [80].

A recent study by Iversen and colleagues revealed that SOD3 can also present in the intravesicular compartment of neutrophils in a resting state and be released as a cargo of EVs when the neutrophil is stimulated by formyl-methionyl-leucyl-phenylalanine or phorbal 12-myristate 13-acetate. The function of SOD3 in EVs is preserved as EVs containing SOD3 can reduce O_2_•^−^ in the extracellular space [96]. Indeed, neutrophils lack SOD3 mRNA. Thus, SOD3 in vesicles might originate from other cells, transfer to neutrophil plasma membranes, and subsequently be stored in endosomes, ready for secretion when needed. However, the original source of transferred SOD3 and the mechanism of how SOD3 is taken up by neutrophils into secretory vesicles have not been investigated. Understanding the mechanism of SOD3 transfer and storage by EVs may help develop a new therapeutic intervention to use EVs as a vehicle to transfer SOD3 to the tissues that require this antioxidant enzyme but have limited capacity to generate their own SOD3.

## 6. Conclusions and Future Direction

Cells release EVs as a compensatory mechanism to remove toxic oxidized protein to maintain homeostasis. Many lines of evidence emphasize the relationship between EVs and oxidative stress. However, the precise mechanisms of how excessive ROS affects the machineries involved in EV biogenesis remain to be elucidated. The study of oxidized molecules in EVs is attractive for novel biomarker discoveries of oxidative stress-related diseases. EV-mediated oxidative stress response could be beneficial or detrimental to the releasing cells. SODs can be transferred via the EV pathway and their antioxidant functions preserved in recipient cells. We predict that overexpression of SOD or treatment with SOD mimetics will prevent the formation of EVs containing oxidative damage markers. It is possible that EVs will serve as sensitive and practical biomarkers to predict antioxidant capacity against chemotherapy of individual patients. Further intensive investigations are needed to decipher the complex relationship between EVs and SOD in order to gain a clearer picture for future therapeutic interventions.

## Figures and Tables

**Table 1:** Role of oxidative stress-related EVs on cell viability and tissue inflammation.

Cell/Tissue Type of Origin	EV Type	Oxidative Stress Condition	Oxidative Stress-Related Cargo	Effect	Reference
Cardiomyocytes	Exosomes	Ethanol, hypoxia/reoxy-genation	HSP60	TLR4-mediated apoptosis	Heiserman et al. [67]
Mast cells	Exosomes	H_2_O_2_	mRNA	H_2_O_2_ tolerance	Eldh et al. [68]
Retinal pigment epithelial cells	Exosomes	Ethanol	VEGF protein and mRNA	Angiogenesis	Atienzar-Aroca et al. [69]
HEK293 cells	Exosomes + MVs	Ca^2+^ ionophore (Lipoxygenase stimulator)	Oxidized phospholipids	TLR4-mediated NFκB activation	Mancek-Keber et al. [70]
Liver	MVs	High fat diet treated mice (NASH model)	Oxidized mtDNA	TLR9-induced TNFα, IL-6 production	Garcia-Martinez et al. [71]
Liver	MVs	Chronic-plus-binge alcohol drinking	mtDNA	TLR9-mediated neutrophilic inflammation	Cai et al. [72]
Liver	Exosomes	Alcoholic hepatitis	miR-122	Sensitize monocytes to LPS	Momen-Heravi et al. [73]
Liver	MVs	Saturated fatty acid-induced lipotoxicity	TRAIL	DR5-dependent macrophage activation	Hirsova et al. [74]
Macrophage	Exosomes	Myocardial infarction	miR-155	Fibroblast inflammation	Wang et al. [75]
Cardiosphere-derived cells	EVs	Myocardial infarction	Y RNA fragment	IL-10 expression and secretion	Cambier et al. [76]
